# Transition from Childhood to Adulthood in Patients with Duchenne Muscular Dystrophy

**DOI:** 10.3390/medicina56090426

**Published:** 2020-08-24

**Authors:** Eliza Wasilewska, Sylwia Małgorzewicz, Agnieszka Sobierajska-Rek, Joanna Jabłońska-Brudło, Lucyna Górska, Karolina Śledzińska, Joanna Bautembach-Minkowska, Jolanta Wierzba

**Affiliations:** 1 Department of Allergology and Pulmonology, Medical University of Gdańsk, 80-211 Gdańsk, Poland; ewasilewska@gumed.edu.pl (E.W.); lucyna.gorska@gumed.edu.pl (L.G.); 2 Department of Clinical Nutrition, Medical University of Gdansk, 80-211 Gdańsk, Poland; 3 Department of Rehabilitation Medicine, Medical University of Gdansk, 80-211 Gdańsk, Poland; sobierajska@gumed.edu.pl (A.S.-R.); Joanna.jablonska-brudlo@gumed.edu.pl (J.J.-B.); 4 Department of Internal and Pediatric Nursing, Institute of Nursing and Midwifery, Medical University of Gdansk, 80-211 Gdańsk, Poland; Karolina.sledzinska@gumed.edu.pl (K.Ś.); Jolanta.wierzba@gumed.edu.pl (J.W.); 5 Department of Pediatrics, Diabetology and Endocrinology, Medical University of Gdańsk, 80-211 Gdańsk, Poland; Joanna.minkowska@gumed.edu.pl

**Keywords:** duchenne muscular dystrophy, transition process, health needs

## Abstract

Recently, progress has been observed in the knowledge about Duchenne Muscular Dystrophy (DMD), which is a severe and commonly diagnosed genetic myopathy in childhood, historically resulting in early death. Currently, there are a lot of methods available to improve the clinical course of DMD and extend patients’ life expectancy to more than 30 years of age. The key issue for DMD patients is the period between 16–18 years of age, which is described as a transition from pediatric- to adult-oriented healthcare. Adolescents and adults with DMD have highly complex healthcare needs associated with long-term steroid usage, orthopedic, ventilation, cardiac, and gastrointestinal problems. The current paper provides a comprehensive overview of special healthcare needs related to the transfer of a patient with DMD from child-oriented to adult-oriented care. Additionally, the need to organize effective care for adults with DMD is presented.

## 1. Introduction

Rare diseases (RDs) are serious, chronic, in most cases progressive, genetic, and life-threatening. RDs currently affect 3.5–5.9% of the population worldwide, which means over 300 million people are living with rare diseases around the world [1,2]. Recently, we have observed huge progress in the medical and scientific knowledge in that area, and nowadays, more than 7500 RDs are known [1,2,3,4].

One of the most severe and commonly diagnosed RDs is Duchenne muscular dystrophy (DMD; OMIM #310200). The etiology of this congenital X-linked disease affecting 1 in 3500 persons, mostly male, are point mutations, deletion, or duplication of gene-coding dystrophin. This is the protein responsible for proper muscle structure by creating an internal fiber cytoskeleton [5,6]. In the clinical course, we observe muscle fiber necrosis, and the replacement of them by fibrosis and connective tissue triggers the disruption of a large number of organs, in particular, the skeletal, heart, and respiratory systems. Besides muscle, there are several other tissue-specific isoforms of dystrophin, some exclusively or predominantly expressed in the brain or the retina, and can be associated with the central nervous system (CNS) involvement that is common in DMD.

Historically, insufficiency in the cardiorespiratory system caused death before 18 years of age [5,7]. Therefore there was no problem with the transition from childhood to adulthood and DMD was considered a “pediatric” disease for a long time. Nowadays, there is still no targeted effective therapy for DMD, but the appropriate treatment and medical care can improve the quality of life of those patients, and extend their life expectancy to more than 30 years of age [5,7,8,9,10,11].

Prolonging the survival of the patients leads to new consequences in several areas in relation to law, education, and social care services. It particularly refers to the transition from pediatric to adult health services [12]. Currently, situations where a patient with DMD in an advanced stage of the disease and with multiple organ dysfunction is transferred from pediatric care to adult care are becoming more frequent.

There are still very few publications describing adult patients with DMD and their health problems related to the progression of the disease during the transition [13,14,15]. The current paper provides a comprehensive overview of special healthcare needs related to the transfer of a patient with DMD from child-oriented to adult-oriented healthcare by consulting the main libraries (Pubmed, Scopus, and Google Scholar). Most recent bibliographical sources have been applied. Additionally, the need to organize effective care for adults with DMD is presented.

## 2. Process of Transition

The transition of DMD patients from pediatric to adult services is often a problematic process; therefore, it should be well planned (Table 1) [12,14,16,17,18]. First, the healthcare professional who attends to the unique challenges of transition should be identified. This person should take responsibility for current healthcare, care coordination, and future healthcare planning [18]. According to the guidelines, the planning of the healthcare transition process should start even from 14 years of age. Then, it is vital to create a written transition plan together with the young person and their family. Positive experiences are characterized by forward-planning and long-standing relationships between the family and healthcare professionals.

For adolescents and young patients with DMD, the transition is a very important time. They lose their mobility. How they will spend this time, and how they will be provided with specialist care is extremely important. When some young people are not transferred from child to adult services with adequate healthcare plans, it can result in their exclusion from adult services [15,19]. For many, this means losing the coordination of treatments between specialists and worsening medical care. This is especially important because it is often time-associated with many difficult decisions regarding invasive treatment, e.g., a tracheostomy tube or percutaneous endoscopic gastrostomy (PEG). The transit from childhood to adulthood is a process and covers many different areas in the life of a DMD patient: health and social care services with relevance to education, law, and psychological support [12,19].

## 3. Healthcare Needs in the Transitional Age

DMD has the following stages: childhood—the appearance of the first symptoms and the diagnosis (2–6 years of age) and increase of clinical symptoms while maintaining of motor independence (6–9 years of age); adolescence—loss of ambulation (10–14 years of age); and adulthood over 15 years of age. The transitional age from childhood to adulthood begins with adolescence and coincides with the loss of independent walking. After that, dysfunction in many organs appears. The milestones in the disease progression are the lack of ambulation, loss of ability to use the upper limbs, gastrointestinal problems resulting in malnutrition, heart failure, and respiratory insufficiency (see Table 2) [5,7,10].

### 3.1. Muscle and Skeletal System—Required Rehabilitation

Progressing muscle weakness is the main manifestation of the disease; however, this is exacerbated into adulthood by a lack of physical activity. Current guidelines recommend regular submaximal activities for patients with DMD during every stage of the disease [5]. Physical training can preserve endurance, functional abilities, and counter muscle atrophy [20]. Most adolescents and adults with DMD are non-ambulant. The predicting factors of loss of ambulation in DMD patients are the loss of standing up from the floor, loss of lie-to-sit ability, loss of stair-climbing ability, and loss of ability to stand up from a chair [21]. For this group of patients, the milestones in the disease progression will consecutively be the loss of ability to turn in bed, loss of head control, loss of trunk mobility, and loss of ability to control a wheelchair [22].

Another problem in DMD patients is contractures. They are associated with loss of joint range of motion (ROM), static positioning, muscle imbalance between agonists and antagonists, and fibrotic changes (fatty tissue infiltration) in the muscles. In ambulant patients, contractures generally affect lower extremities—hips, knees, ankle joints, iliotibial band, hamstrings, and gastrocnemius—and start to develop in elbows, wrists, and long finger flexors after the loss of independent ambulation [23]. The management of contractures should consist of regular stretching, night splints, and maintaining an upright position with the use of a standing device. Daytime orthoses (AFO—ankle-foot orthoses) should be considered in the early non-ambulatory stage and be worn for most of the day. Besides preventing the increase of feet deformity, AFOs also improve postural control and stability. Study results of contracture management in DMD support the need for comprehensive individualized approaches consisting of optimal positioning, individualized use of splinting, orthotic interventions, standing devices, serial casting, and custom seating and power positioning components in mobility devices [5,24,25]. When hand contractures develop, resting hand splints may be considered [26].

Preserving good upper limb function is essential for providing independence in non-ambulant DMD patients. There is a direct relationship between arm function and the performance of activities, such as feeding, bathing, brushing teeth, and using powered devices or a computer [27]. The milestones in disease progression for the upper extremities are the loss of ability to reach over the head, loss of ability to reach the scalp, loss of ability to self-feed without adaptations (hand to mouth), loss of ability to place hands on a tabletop, and loss of ability to use a computer (distal hand function) [28]. When problems with self-feeding appear, the use of an upper-limb-powered support device should be considered.

With the loss of independent ambulation and spending most of the time sitting, a change of spine curvature develops: lumbar kyphotization and the progressing weakness of the paraspinal muscles lead to scoliosis [29]. During deterioration of the patient’s condition, scoliosis is progressing. A massive deformity determines the risk of cardiorespiratory dysfunction. With 10 degrees of thoracic curve progression, functional vital capacity can decrease by 4% [30]. This deformity may also cause costo-iliac impingement, pain, development of pressure ulcers, and nursery problems. Rapidly progressing scoliosis makes sitting difficult, impacts the use of upper limbs, and significantly deteriorates the quality of life of DMD patients. Unfortunately, there is no evidence that using standing frames or customized wheelchairs prevents scoliosis [31,32]. However, the constant management of wheelchair size, cushion support, and proper seating, as well as exercises activating the trunk muscles and the learning of postural self-correction, may slow down the scoliosis progression. In the late stages, head support in the wheelchair is essential and can slow down the progression of scoliosis [33]. For progressive scoliosis in the late non-ambulatory stage of the disease, posterior spinal fusion is recommended [5].

Respiratory management (chest physiotherapy) is a key component of respiratory care for DMD. In rehabilitation, a cough assistance device is also used.

### 3.2. Respiratory Support

Obstructive sleep apnea (OSA) and hypoventilation (daytime drowsiness, distraction, chronic headaches, disorders of concentration, difficulty falling asleep and waking up, awakening during the night, nightmares, and aphorism) occur more often as the disease progresses. In adolescents, nocturnal hypercapnia may occur as the first sign of respiratory insufficiency. Additionally, adult DMD patients, particularly those with a forced vital capacity (FVC) < 60%, non-invasive ventilation, presence of a tracheostoma, weak cough, and weak airway clearance or cardiomyopathy, are at high risk of severe respiratory infection [10,11,34].

Assessment and supportive treatment of the pulmonary function is one of the most important issues in patients with DMD. Spirometry is recommended at least once a year, and after the loss of ambulance, at least every 6 months with a special assessment of FVC and peak expiratory flow (PEF), which show progressive restriction [11,35].

Measurements of cough peak flow (CPF), muscle strength, maximal inspiratory/expiratory pressures (MIP/MEP) through the mouth, and sniff nasal pressure inspirations (SNIP) through the nose are also useful [34,35,36]. Polysomnography (PSG) should be performed in all patients with OSA symptoms, and with hypoventilation or respiratory failure [37,38]. Respiratory rehabilitation and improvement of the cough reflex are indicated by hand or mechanically from an early stage of the disease [36,39]. Afterward, it is necessary to suck discharge from the respiratory tract through suction. In advanced stages of the disease, a small respiratory infection can cause respiratory failure. Then, patients require breathing support, such as non-invasive mechanical ventilation (NIV) or sometimes even through a tracheostomy tube [40].

Patients without a respiratory infection but with symptoms of hypoventilation also should use NIV [11,35]. Studies have shown that the average age of inclusion of NIV into treatment was around 18 years [37,38]. The American Thoracic Society recommends considering NIV when symptoms of hypoventilation and hypercapnia occur, and FVC decreases below 50% of the predicate volume [36]. Initially, NIV is used periodically only at night to relieve night hypoventilation. NIV during the day is recommended if hypercapnia (Pco_2_ < 50 mm Hg) or low saturation (So_2_ < 92%) occurs [39]. Positive airway pressure (PAP) is preferred in the treatment of OSA, and biphasic or bilevel positive airway pressure (BIPAP) is preferred when respiratory failure occurs [40,41]. It is a long-term therapy, and in the era of telemedicine, can be safely conducted in the patient’s home [42].

In the case of NIV ineffectiveness, patients require a tracheostomy and mechanical ventilation [43]. Abnormal arteriovenous or venous gasometry and respiratory acidosis are late but important signals indicating the need for assisted ventilation [36]. The decision to implement a tracheostomy tube should be made together with the patient and his caregivers.

### 3.3. Cardiological Care

Defects of the structure and function of cardiomyocytes and the replacement of muscle tissue with fibrous and fatty tissue result in symptoms of heart dysfunction. Multiple fibrosis is observed within the lower-lateral wall of the heart, gradually covering the whole heart [44].

Initially, there are clinically asymptomatic changes in the electrocardiography (ECG) and subclinical impairment of diastolic function, then widening of the heart cavity, worsening of systolic function, dilated cardiomyopathy, and eventually extreme heart failure [45]. Heart failure can be presented in more than 80% of patients over 18 years of age and accounts for up to 40% of deaths [10,11].

Asymptomatic patients are recommended to have ECG and echocardiography (ECHO) control and cardiac consultation once a year. Patients with abnormal cardiac tests should be monitored every 6 months, or more often according to cardiologists, and have Holter ECG examinations every 12 months [10].

Magnetic resonance imaging of the heart (CMR) is becoming increasingly important today and it is even the procedure of choice in the cardiac diagnosis in DMD in the United States. CMR is a well-established and reliable tool for early and sensitive diagnosis of cardiac involvement, mainly due to the possibility of a non-invasive myocardial tissue characterization with the detection of even subtle forms of myocardial fibrosis via late gadolinium enhancement (LGE)-imaging or T1-mapping techniques as reliable tools for the early and sensitive diagnosis of cardiac involvement [10]. This method assesses the presence and extent of the foci of fibrosis in the heart. In addition, CMR depicts the heart in patients with worse tissue translucency or significant deformation of the chest, which narrows the acoustic window. In the advanced phase of the disease, poor control of breath by the patient results in a worse quality of the obtained images.

The purpose of cardiac treatment of patients with DMD is to delay the appearance of cardiomyopathy and heart failure, and in patients with reduced contraction of the ventricles of the heart chamber, slowing the progression of the disease and reducing the severity of symptoms. It is currently recommended to already start therapy with angiotensin convertase inhibitors (ACEi) or a beta-blocker in patients with reduced contraction during asymptomatic periods. Further treatment is tailored to the individual, depending on the course of the disease and the occurring symptoms, and remains the decision of the leading cardiologist [46,47,48].

### 3.4. Gastrointestinal and Nutritional Aspects

Recommendations from the 2018 DMD Care Considerations include a registered dietitian nutritionist (RDN) as an essential member of the multidisciplinary DMD care team, which consists of a pediatrician, general practitioner, pulmonologist, cardiologist, physical and rehabilitation medicine specialist, orthopedist, physiotherapist, nutritionist, psychologist, and speech therapist [5]. Due to a number of gastroenterological problems, the dietitian’s help is highly recommended throughout the illness. At the beginning of the disease, overweight and obesity are basic problems. Steroid therapy, immobility, and reduced energy expenditure result in a higher risk of developing obesity and a metabolic syndrome with insulin resistance, hyperglycemia, and visceral obesity. Gastroenterological disorders, such as gastroesophageal reflux, gastric emptying, and constipation may then be diagnosed. As the disease persists, swallowing disorders appear, followed by dysphagia. Initial dietary recommendations that focus on the fight against excess body mass change because the main goal of nutrition in adulthood is to fight malnutrition. As dysphagia progresses, calorie intake decreases and unintentional, progressive weight loss is typical. Malnutrition can also occur when patients lose the ability to self-feed, for example, when progressive weakness in the upper limbs or postoperative complications after spinal fusion surgery occur [49].

However, orofacial problems among patients with DMD, such as with eating, speaking, facial and tongue movements, and changes of the tongue and teeth, are self-reported by up to approx. 70% of patients. In our opinion, oral surgery is the last therapeutic option. The guidelines should emphasize the importance of referral to a speech therapist and the role of food adjustment from solid to semi-liquid and flushing with liquid [5].

After swallowing disorders have been diagnosed, a dietitian should propose changing the diet plan, i.e., modifying the consistency, quantity, and volume of meals, and using food thickeners recommended for dysphagia. The next step is to enrich the oral diet through the supply of dietary supplements (ONS). ONS in liquid or powder form can provide an additional 30 g of protein and about 600 kcal/day, along with a complete set of vitamins and microelements [50].

The time of the qualification for artificial nutrition is not set; in practice, decisions should be made individually after discussing all the benefits with the patient and their family.

According to generally recommended standards, if the patient is malnourished, if they do not consume at least 50% of the daily demand for calories and other nutrients, and the process is progressive, they should qualify for nutrition via PEG. Qualification to enteral nutrition can take place in the hospital, as well as in the home nutrition center or at the patient’s home. PEG feeding is provided at home, but the patient should be under the care of a home nutrition center. During the initial educational meeting, the indications and goals of the treatment, care of the access to the gastrointestinal tract, and diet supply are discussed [51].

Properly conducted education allows for avoiding a number of complications, enables efficient communication with home nutrition center staff, and the early identification of problems [52].

### 3.5. Endocrinology Problems

Low growth, delayed sexual adolescence, osteoporosis, and suppression of the adrenal cortex are often diagnosed in patients with DMD. They result from both the disease and the complications of steroid therapy [5].

The slower rate of growth already occurs in the first years of life. Then, it oscillates around the lower centile values, and, after adolescence, the final growth is below the third centile in most patients [53,54,55,56].

Delayed sexual maturation occurs in between 50–100% of patients with DMD treated with steroids, and is caused by inhibition by steroids of the hypothalamic–pituitary–gonad axis and the development of hypogonadotropic hypogonadism. Confirmation of hypogonadism is an indication for testosterone treatment in boys over 14 years of age in whom features of sexual maturation are not found [5,10].

Osteoporosis in DMD is due to chronic steroid therapy, reduced mobility of patients, weakened muscle strength, delayed sexual adolescence, and nutritional problems. Clinical symptoms of osteoporosis include chronic bone pain, vertebral fractures (approx. 40% asymptomatic) or long bones, decreased body height, and bone deformations. Densitometry (DXA) is commissioned before starting steroid therapy, then a check is done every 1–2 years, depending on the risk factors. Side X-rays of the spine during steroid therapy, if kyphoscoliosis or persistent back pain is present, should also be performed. Calcium phosphate management should be monitored, and vitamin D3 and calcium supplementation should be taken into account in the diet. Treatment with intravenous bisphosphonates should be considered after the confirmation of osteoporosis [10,57].

Nowadays, effective therapy for DMD is unavailable; steroids are the drug of choice. Hypothalamic–pituitary–gonad (HPG) axis suppression can be the result if the dose of prednisone exceeds 3 mg/m^2^/day. After the discontinuation of steroids, resumption of cortisol production through the adrenal glands can take 6–12 months, depending on the dose, method of administration, and duration of the steroid therapy. The discontinuation of steroids should take place gradually due to the risk of developing adrenal insufficiency and shock [5,10]. It is recommended that stressful doses of steroids are administered during the perioperative period and acute disease in patients with an inhibited HPG axis. Adrenal failure is a life-threatening condition with alarming symptoms, such as fatigue, apathy, loss of appetite, headaches, nausea, vomiting, abdominal pain, low blood pressure, fainting, and loss of consciousness [58].

### 3.6. Nephrological Problems

The incidence of genitourinary (GU) conditions may increase from 8% up to 27% at the age of 30. The most common reason for GU disorders is voiding dysfunction, followed by GU tract infection and kidney/ureter calculus. Disease progression, manifested as a loss of ambulation, is associated with a significantly increased risk of the onset of GU disorders. Furthermore, the renal failure marker cystatine C should be monitored in patients older than 20 years of age with DMD; the association between cardiac and renal dysfunction in patients with advanced-stage DMD was described [11].

### 3.7. Orthopedic Surgery

Qualification for orthopedic surgery due to scoliosis is very difficult and must be performed in a specialized center that is experienced in this type of surgery. Surgery is recommended when the degree of scoliosis (known as the Cobb angle) is greater than 20 degrees in boys who are still growing and who are not taking steroids. The aim of the operation is to maintain the best possible posture for the child’s fitness and comfort. When boys take steroids, the risk of deterioration is lower; therefore, the decision to operate may be postponed until the Cobb angle is greater than 40 degrees [58].

### 3.8. Mental Health

#### 3.8.1. Patients

The variable expression of dystrophin isoforms in the brain of boys with DMD in relation to the type and locus of the mutation probably leading to abnormalities on the central nervous system (CNS) can be found in MRI scans [58].

The patients with DMD experienced intellectual disability (7–27% of patients) and learning difficulties: language-based skills, such as reading (26%); attention-deficit hyperactivity disorder (ADHD; 32%); autism spectrum disorders (ASDs; 15%); and anxiety (27%). Despite this, almost all of them completed primary school, 23% completed high school, and 23% started vocational education with 9% completing it [59].

Neuropsychological screening is recommended at the time of the diagnosis, then at every neuromuscular clinic visit or optionally every 2–3 years, or at any time of the crisis or loss of independence in daily activities, loss of ambulatory status, the onset of feeding difficulties or ventilatory support, and the transition to the adult healthcare system. If any abnormalities in neuropsychological development are found, prompt psychological or psychiatric consultation is needed, with a further decision regarding the type of therapy or medication needed. Moreover, screening for hearing and vision impairment should be performed to prevent secondary intellectual disabilities.

Despite the higher incidence of neuropsychological disorders, according to a study performed by Rahbek et al. [60], adult DMD patients state their quality of life (QoL) as excellent. Most of them have a positive experience with personal assistance, education, housing, financial situation, and immobilization. They claim that they are capable of participating in a variety of activities typical of a normal lifestyle. However, DMD patients complain of inadequate education, love life, and the increased frequency of pains. It is interesting that in children with DMD, virtually all aspects of the health-related quality of life (HRQoL) were significantly impaired when compared with published normative data for boys with other chronic illnesses, while adolescents and adults with DMD did not differ from published normative data in psychosocial areas of HRQoL, despite significant reductions in the physical aspects of HRQoL [61]. Further studies describe their psychosocial QoL as lower than in the general pediatric population, with loss of ambulation as the most depressing factor, but surprisingly increasing age or corticosteroid use has little impact on general well-being [61]. A lower QoL is stated in patients from Eastern European countries compared to Western countries, which could be explained by the lower socio-economic status of these patients. Patients with DMD should have adequate psychosocial care provided throughout the whole life.

#### 3.8.2. Caregiver Burden

Caregiving in DMD was reported as a subjective substantial burden, frequently associated with impaired HRQoL, increased incidence of depression and anxiety, family dysfunction, stress, pains, lower self-esteem, poor sleep quality, and sexual dysfunction [62].

Regarding siblings’ involvement, half of them declare “a great deal” or at least “some” commitment to the DMD patient care, whereas the HRQoL and psychological functioning of siblings is comparable with the general population, with a higher tendency toward emotional problems. It is also noticeable in the DMD families that carers stop working or reduce working hours to optimally manage their child; it may cause difficulties at work, in taking holidays, and neglecting their hobbies [63,64].

Although many carers suffer from depression and anxiety (20–50%), the care of a DMD patient by the majority of carers (66%) has an overall positive impact on their life [63]. In their study, Pangalila et al. reported that almost everyone (96%) enjoyed the caregiving, and siblings declared the strengthening of family bonds, increased knowledge, and maturity [65].

To better deal with everyday life difficulties of care, siblings are advised to get connected with other siblings of DMD boys through friends, websites, or social media. It is also important to ensure respite care for a family or caregivers for weekends or holidays, or, if affordable, personal care attendants.

### 3.9. Palliative Care

During the transition period, the issue of long-term care goals should be raised. Concerns that discussions about the progression of the disease not taking place early enough have been reported [13]. Adults with DMD are open to a discussion regarding topics such as death and dying (e.g., the nature and place of death; practical planning for funerals and wills; how they prefer to be comforted, supported, treated, and remembered). Very often, no emotional or psychological support to think about the end of life is routinely offered and patients find it very difficult to discuss these issues with family members [19].

Although cardiorespiratory failure is the main cause of death among DMD patients, premature death may appear in patients with no previous history of cardiorespiratory disorders. Factors contributing to premature death are respiratory failure, inadequate nutrition, non-attendance at appointments, suboptimal coordination of care, decreased psychological wellbeing, fat embolism, cardiac arrhythmia, and adrenal insufficiency [66].

## 4. Summary and Conclusions

Nowadays, increasingly more people with DMD are living longer and reach the age of 30. This leads to many different challenges in some areas of their functioning. One of the fundamental issues is the transfer of DMD patients from child to adult healthcare services. Adolescents and adults with DMD have complex healthcare needs associated with the progression of the disease. The most important are the weakness of muscle, resulting in the immobilization of lower, then upper, limbs; breathing support; treatment of cardiac complications (cardiomyopathy, arrhythmia); and gastroenterological failure, resulting in malnutrition. The transition should be a well-planned and coordinated process and should start as early as the adolescent period. The goal is to maximize lifelong functioning through the appropriate healthcare services that continue uninterrupted as the individual moves from adolescence to adulthood. Specialists should have adequate knowledge about this disease, and be constantly assigned to patients with special health needs in their centers. Such a care system, called a “Centre of Excellence,” is currently the most optimal in the treatment of rare diseases. A multidisciplinary team should be involved in the transition process. Due to the health problems that increase with age, an array of specialists is necessary, such as a pediatrician, general practitioner, pulmonologist, cardiologist, physical and rehabilitation specialist, orthopedist, nutritionist, psychologist, and speech therapist. It is advisable to properly organize a center dedicated to adult DMD patients.

## Figures and Tables

**Table 1:** Steps in the transition from child-oriented to adult-oriented healthcare (modified from The American Academy of Pediatrics and American Society of Internal Medicine consensus [18]).

Steps in the Transition	
1.	Identify a healthcare professional who attends to the care coordination and future healthcare planning
2.	Identify the core knowledge and skills required to provide developmentally appropriate healthcare transition services (rehabilitation, pulmonology, cardiology, gastrointestinal, and nutrition care)
3.	Prepare a medical summary that provides the common knowledge base for collaboration between healthcare professionals
4.	Create a written healthcare transition plan by age 14 together with the young person and family
5.	Apply the same guidelines for primary and preventive care for all adolescents and young adults
6.	Ensure affordable, continuous health insurance coverage with the healthcare transition planning and care coordination for those who have complex medical conditions.

**Table 2:** Special health needs in patients with Duchenne muscular dystrophy.

	Childhood (2–10 Years)	Adolescent (12–15 Years)	Young Adult (>15 Years)
Neurology	Ambulatory	Ambulatory/non-ambulatory	Non-ambulatory
Rehabilitation	Recreational activities, sub-maximal aerobic exercise, stretching of lower limbs	Hydrotherapy/swimming, stretching of lower and upper limbs, postural advice (seating, standing frames, wheelchairs), orthotics, sub-maximal aerobic exercise (i.e., assisted cycling), rehabilitation of the cough reflex using a cough assistance device	Powered wheelchairs, positioning, stretching, sub-maximal aerobic exercise (i.e., arm ergometer), respiratory management (chest physiotherapy), rehabilitation of the cough reflex using a cough assistance device
Respiratory system	Vaccinations (pneumococcus) Spirometry, MEP, MIP, polysomnography	Hypoventilation, weakness cough reflex, OSA spirometry, MEP, MIP, oxidation; rehabilitation to strengthen the cough reflex, NIV	Respiratory failure, aspiration and respiratory infections, gasometry, oxidation NIV, mechanical ventilation
Gastrointestinal	Normal	GER, constipation	Swallowing problems, GER, constipation, gastrointestinal dilation, alleged intestinal obstruction
Nutrition	Recommendations for healthy children and the prevention of obesity	Recommendations for healthy children and the prevention of obesity and malnutrition	Qualification for artificial nutrition (PEG)
Endocrinology	Low growth	Low growth, osteoporosis (calcium, vitamin D supplementation), delayed sexual adolescence	Low growth, osteoporosis, suppression of the adrenal cortex
Cardiology	Normal	Cardiomyopathy, arrhythmias	Cardiomyopathy, arrhythmias
Nephrology	Normal	Voiding dysfunction, followed by a GU tract infection and kidney/ureter calculus	Voiding dysfunction, GU tract infection and kidney/ureter calculus
Mental health	Neuropsychological screening, ADHD, ASD	Neuropsychological screening intellectual disability, ADHD, ASD, anxiety	Neuropsychological screening, intellectual disability, ASD, anxiety
Psychosocial	Normal	Missed activities and friends, inadequate education, pains	Inadequate education, lack of love life, the increased frequency of pains, fatigue, isolation

GER, gastric esophagus reflux; MEP, maximal expiratory pressures; MIP, maximal inspiratory pressures; NIV, non-invasive ventilation; OSA, obstructive sleep apnea; PEG, percutaneous endoscopic gastrostomy; ADHD, attention-deficit hyperactivity disorder; ASD, autism spectrum disorders; GU, genitourinary.

## References

[1] Wakap S.N., Lambert D.M., Olry A., Rodwell C., Gueydan C., Lanneau V., Murphy D., Le Cam Y., Rath A. (2019). Estimating cumulative point prevalence of rare diseases: Analysis of the Orphanet database. Eur. J. Hum. Genet..

[2] https://www.orpha.net/consor/cgi-bin/index.php.

[3] Valdez R., Ouyang L., Bolen J. (2016). Public Health and Rare Diseases: Oxymoron No More. Prev. Chronic Dis..

[4] https://www.eurordis.org/IMG/pdf/princeps_document-EN.pdf.

[5] Birnkrant D.J., Bushby K., Bann C.M., Apkon S.D., Blackwell A., Brumbaugh D., E Case L., Clemens P.R., Hadjiyannakis S., Pandya S. (2018). Diagnosis and management of Duchenne muscular dystrophy, part 1: Diagnosis, and neuromuscular, rehabilitation, endocrine, and gastrointestinal and nutritional management. Lancet Neurol..

[6] Hoffman E.P., Brown R.H., Kunkel L.M. (1987). Dystrophin: The protein product of the duchenne muscular dystrophy locus. Cell.

[7] Koeks Z., Bladen C.L., Salgado D., Van Zwet E., Pogoryelova O., McMacken G., Monges S., Foncuberta M.E., Kekou K., Kosma K. (2017). Clinical Outcomes in Duchenne Muscular Dystrophy: A Study of 5345 Patients from the TREAT-NMD DMD Global Database. J. Neuromuscul. Dis..

[8] Emery A.E. (1991). Population frequencies of inherited neuromuscular diseases—A world survey. Neuromuscul. Disord..

[9] Roberto R., Fritz A., Hagar Y., Boice B., Skalsky A., Hwang H., Beckett L.A., McDonald C.M., Gupta M. (2011). The Natural History of Cardiac and Pulmonary Function Decline in Patients with Duchenne Muscular Dystrophy. Spine.

[10] Birnkrant D.J., Bushby K., Bann C.M., Alman B.A., Apkon S.D., Blackwell A., Case L.E., Cripe L., Hadjiyannakis S., Olson A.K. (2018). Diagnosis and management of Duchenne muscular dystrophy, part 2: Respiratory, cardiac, bone health, and orthopaedic management. Lancet Neurol..

[11] Bushby K., Finkel R., Birnkrant D.J., Case L.E., Clemens P.R., Cripe L., Kaul A., Kinnett K., McDonald C.M., Pandya S. (2010). Diagnosis and management of Duchenne muscular dystrophy, part 2: Implementation of multidisciplinary care. Lancet Neurol..

[12] Rodger S., Steffensen B.F., Lochmuller H. (2012). Transition from childhood to adulthood in Duchenne muscular dystrophy (DMD). Orphanet J. Rare Dis..

[13] Colvin M.K., Poysky J., Kinnett K., Damiani M., Gibbons M., Hoskin J., Moreland S., Trout C.J., Weidner N. (2018). Psychosocial Management of the Patient with Duchenne Muscular Dystrophy. Pediatrics.

[14] https://research-information.bris.ac.uk/en/projects/transition-to-adulthoodthe-experience-and-needs-of-young-men-with.

[15] Schrans D., Abbott D., Peay H., Pangalila R., Vroom E., Goemans N., Vles J., Aldenkamp A., Hendriksen J. (2013). Transition in Duchenne Muscular Dystrophy: An expert meeting report and description of transition needs in an emergent patient population: (Parent Project Muscular Dystrophy Transition Expert Meeting 17–18 June 2011, Amsterdam, The Netherlands). Neuromuscul. Disord..

[16] Rosen D.S., Blum R.W., Britto M., Sawyer S.M., Siegel D.M. (2003). Transition to adult health care for adolescents and young adults with chronic conditions: Position paper of the Society for Adolescent Medicine. J. Adolesc. Health.

[17] https://www.gottransition.org/.

[18] American Academy of Pediatrics, American Academy of Family Physicians, American College of Physicians-American Society of Internal Medicine (2002). A consensus statement on health care transitions for young adults with special health care needs. Pediatrics.

[19] Abbott D., Prescott H., Forbes K., Fraser J., Majumdar A. (2017). Men with Duchenne muscular dystrophy and end of life planning. Neuromuscul. Disord..

[20] Landisch R.M., Kosir A.M., Nelson S.A., Baltgalvis K.A., Lowe D.A. (2008). Adaptive and nonadaptive responses to voluntary wheel running by mdx mice. Muscle Nerve.

[21] Scott E., Eagle M., Mayhew A., Freeman J., Main M., Sheehan J., Manzur A.Y., Muntoni F., North Star Clinical Network for Paediatric Neuromuscular Disease (2011). Development of a Functional Assessment Scale for Ambulatory Boys with Duchenne Muscular Dystrophy. Physiother. Res. Int..

[22] Steffensen B.F., Hyde S., Lyager S., Mattsson E. (2001). Validity of the EK scale: A functional assessment of non-ambulatory individuals with Duchenne muscular dystrophy or spinal muscular atrophy. Physiother. Res. Int..

[23] Kiefer M., Bonarrigo K., Quatman-Yates C., Fowler A., Horn P.S., Wong B.L. (2019). Progression of Ankle Plantarflexion Contractures and Functional Decline in Duchenne Muscular Dystrophy: Implications for Physical Therapy Management. Pediatr. Phys. Ther..

[24] Carroll K., De Valle K., Kornberg A., Ryan M., Kennedy R.A. (2017). Evaluation of Serial Casting for Boys with Duchenne Muscular Dystrophy: A Case Report. Phys. Occup. Ther. Pediatr..

[25] Nishizawa H., Matsukiyo A., Shiba N., Koinuma M., Nakamura A. (2018). The effect of wearing night splints for one year on the standing motor function of patients with Duchenne muscular dystrophy. J. Phys. Ther. Sci..

[26] Weichbrodt J., Eriksson B.-M., Kroksmark A.-K. (2017). Evaluation of hand orthoses in Duchenne muscular dystrophy. Disabil. Rehabil..

[27] Connolly A.M., Malkus E.C., Mendell J.R., Flanigan K.M., Miller J.P., Schierbecker J.R., Siener C.A., Golumbek P.T., Zaidman C.M., McDonald C.M. (2015). Outcome reliability in non-Ambulatory Boys/Men with duchenne muscular dystrophy. Muscle Nerve.

[28] Mayhew A., Coratti G., Mazzone E.S., Klingels K., James M., Pane M., Straub V., Goemans N., Mercuri E., Ricotti V. (2019). Performance of Upper Limb module for Duchenne muscular dystrophy. Dev. Med. Child Neurol..

[29] Kinali M., Main M., Mercuri E., Muntoni F. (2007). Evolution of abnormal postures in Duchenne muscular dystrophy. Ann. Indian Acad. Neurol..

[30] Choi Y.-A., Shin H.I., Shin H.I. (2019). Scoliosis in Duchenne muscular dystrophy children is fully reducible in the initial stage, and becomes structural over time. BMC Musculoskelet. Disord..

[31] Garg S. (2016). Management of scoliosis in patients with Duchenne muscular dystrophy and spinal muscular atrophy: A literature review. J. Pediatr. Rehabil. Med..

[32] Lebel D.E., Corston J.A., McAdam L.C., Biggar W.D., Alman B.A. (2013). Glucocorticoid Treatment for the Prevention of Scoliosis in Children with Duchenne Muscular Dystrophy: Long-Term Follow-up. J. Bone Jt. Surg. Am. Vol..

[33] Vorster N., Evans K., Murphy N., Kava M.P., Cairns A., Clarke D., Ryan M.M., Siafarikas A., Rowe P.W., Parkinson S. (2019). Powered standing wheelchairs promote independence, health and community involvement in adolescents with Duchenne muscular dystrophy. Neuromuscul. Disord..

[34] Aliverti A., LoMauro A., D’Angelo M.G. (2015). Assessment and management of respiratory function in patients with Duchenne muscular dystrophy: Current and emerging options. Ther. Clin. Risk Manag..

[35] Mayer O.H., Finkel R., Rummey C., Benton M., Glanzman A., Flickinger J., Lindstrom B.-M., Meier T. (2015). Characterization of pulmonary function in Duchenne Muscular Dystrophy. Pediatr. Pulmonol..

[36] Finder J.D. (2009). A 2009 Perspective on the 2004 American Thoracic Society Statement, “Respiratory Care of the Patient with Duchenne Muscular Dystrophy”. Pediatrics.

[37] Hukins C.A., Hillman D.R. (2000). Daytime Predictors of Sleep Hypoventilation in Duchenne Muscular Dystrophy. Am. J. Respir. Crit. Care Med..

[38] Hoque R. (2016). Sleep-Disordered Breathing in Duchenne Muscular Dystrophy: An Assessment of the Literature. J. Clin. Sleep Med..

[39] https://erj.ersjournals.com/content/48/suppl_60/PA3067.

[40] Edwards C., Davies P., Davidson S. (2014). Determining the manner in which patients with Duchenne muscular dystrophy start non-invasive ventilation. Eur. Respir. J..

[41] Santos D.B., Vaugier I., Boussaïd G., Orlikowski D., Prigent H., Lofaso F. (2016). Impact of Noninvasive Ventilation on Lung Volumes and Maximum Respiratory Pressures in Duchenne Muscular Dystrophy. Respir. Care.

[42] Crimi C., Pierucci P., Carlucci A., Cortegiani A., Gregoretti C. (2019). Long-Term Ventilation in Neuromuscular Patients: Review of Concerns, Beliefs, and Ethical Dilemmas. Respiration.

[43] Lyager S., Steffensen B.F., Juhl B. (1995). Indicators of Need for Mechanical Ventilation in Duchenne Muscular Dystrophy and Spinal Muscular Atrophy. Chest.

[44] Verhaert D., Richards K., Rafael-Fortney J.A., Raman S.V. (2011). Cardiac involvement in patients with muscular dystrophies: Magnetic resonance imaging phenotype and genotypic considerations. Circ. Cardiovasc. Imaging.

[45] D’Amario D., Amodeo A., Adorisio R., Tiziano F.D., Leone A.M., Perri G., Bruno P., Massetti M., Ferlini A., Pane M. (2017). A current approach to heart failure in Duchenne muscular dystrophy. Heart.

[46] Spurney C.F., McCaffrey F.M., Cnaan A., Morgenroth L.P., Ghelani S.J., Gordish-Dressman H., Arrieta A., Connolly A.M., Lotze T.E., McDonald C.M. (2015). Feasibility and Reproducibility of Echocardiographic Measures in Children with Muscular Dystrophies. J. Am. Soc. Echocardiogr..

[47] Schram G., Fournier A., LeDuc H., Dahdah N., Thérien J., Vanasse M., Khairy P. (2013). All-Cause Mortality and Cardiovascular Outcomes with Prophylactic Steroid Therapy in Duchenne Muscular Dystrophy. J. Am. Coll. Cardiol..

[48] Finsterer J., Cripe L. (2014). Treatment of dystrophin cardiomyopathies. Nat. Rev. Cardiol..

[49] Brumbaugh D., Watne L., Gottrand F., Gulyas A., Kaul A., Larson J., Tomezsko J. (2018). Nutritional and Gastrointestinal Management of the Patient with Duchenne Muscular Dystrophy. Pediatrics.

[50] Toussaint M., Davidson Z., Bouvoie V., Evenepoel N., Haan J., Soudon P. (2016). Dysphagia in Duchenne muscular dystrophy: Practical recommendations to guide management. Disabil. Rehabil..

[51] Burgos R., Bretón I., Cereda E., Desport J.C., Dziewas R., Genton L., Gomes F., Jésus P., Leischker A., Muscaritoli M. (2018). ESPEN guideline clinical nutrition in neurology. Clin. Nutr..

[52] Mizuno T., Komaki H., Sasaki M., Takanoha S., Kuroda K., Kon K., Mamiya S., Yoshioka M., Yatabe K., Mikata T. (2012). Efficacy and tolerance of gastrostomy feeding in Japanese muscular dystrophy patients. Brain Dev..

[53] Wood C.L., Straub V., Guglieri M., Bushby K., Cheetham T. (2015). Short stature and pubertal delay in Duchenne muscular dystrophy. Arch. Dis. Child..

[54] Rapisarda R., Muntoni F., Gobbi P., Dubowitz V. (1995). Duchenne muscular dystrophy presenting with failure to thrive. Arch. Dis. Child..

[55] Nagel B.H., Mortier W., Elmlinger M., Wollmann H.A., Schmitt K., Ranke M.B. (1999). Short stature in Duchenne muscular dystrophy: A study of 34 patients. Acta Paediatr..

[56] Bishop N., Arundel P., Clark E., Dimitri P., Farr J., Jones G., Mäkitie O., Munns C.F., Shaw N. (2014). Fracture Prediction and the Definition of Osteoporosis in Children and Adolescents: The ISCD 2013 Pediatric Official Positions. J. Clin. Densitom..

[57] Kinnett K., Noritz G. (2017). The PJ Nicholoff Steroid Protocol for Duchenne and Becker Muscular Dystrophy and Adrenal Suppression. PLoS Curr..

[58] Rahbek J., Steffensen B.F., Bushby K., De Groot I.J. (2015). 206th ENMC International Workshop: Care for a novel group of patients—Adults with Duchenne muscular dystrophy Naarden, The Netherlands. Neuromuscul. Disord..

[59] Muntoni F., Torelli S., Ferlini A. (2003). Dystrophin and mutations: One gene, several proteins, multiple phenotypes. Lancet Neurol..

[60] Rahbek J., Werge B., Madsen A., Marquardt J., Steffensen B.F., Jeppesen J. (2005). Adult life with Duchenne muscular dystrophy: Observations among an emerging and unforeseen patient population. Pediatr. Rehabil..

[61] Schmid J., Lutz S., Geers B., Schara U., Elsenbruch S. (2013). Self-Reported Quality of Life and Depressive Symptoms in Children, Adolescents, and Adults with Duchenne Muscular Dystrophy: A Cross-Sectional Survey Study. Neuropediatrics.

[62] Uttley L., Carlton J., Woods H.B., Brazier J.E. (2018). A review of quality of life themes in Duchenne muscular dystrophy for patients and carers. Health Qual. Life Outcomes.

[63] Landfeldt E., Edström J., Buccella F., Kirschner J., Lochmuller H. (2018). Duchenne muscular dystrophy and caregiver burden: A systematic review. Dev. Med. Child Neurol..

[64] Magliano L., Politano L. (2016). Family context in muscular dystrophies: Psychosocial aspects and social integration. Acta Myol..

[65] Pangalila R.F., van den Bos G.A., Bartels B., Bergen M., Stam H.J., Roebroeck M.E. (2015). Prevalence of Fatigue, Pain, and Affective Disorders in Adults with Duchenne Muscular Dystrophy and Their Associations with Quality of Life. Arch. Phys. Med. Rehabil..

[66] Van Ruiten H., Bettolo C.M., Cheetham T., Eagle M., Lochmuller H., Straub V., Bushby K., Guglieri M. (2016). Why are some patients with Duchenne muscular dystrophy dying young: An analysis of causes of death in North East England. Eur. J. Paediatr. Neurol..

